# Radiation-induced alterations in immunogenicity of a murine pancreatic ductal adenocarcinoma cell line

**DOI:** 10.1038/s41598-020-57456-2

**Published:** 2020-01-20

**Authors:** Philipp Schröter, Laura Hartmann, Wolfram Osen, Daniel Baumann, Rienk Offringa, David Eisel, Jürgen Debus, Stefan B. Eichmüller, Stefan Rieken

**Affiliations:** 1 German Cancer Research Center (DKFZ), Research Group GMP & T Cell Therapy, Im Neuenheimer Feld 280, D-69120 Heidelberg, Germany; 2 University Hospital Heidelberg, Department of Radiation Oncology, Im Neuenheimer Feld 400, D-69120 Heidelberg, Germany; 3 Heidelberg Institute of Radiation Oncology (HIRO), Im Neuenheimer Feld 280, D-69120 Heidelberg, Germany; 4 Heidelberg Ion-Beam Therapy Center (HIT), Im Neuenheimer Feld 450, D-69120 Heidelberg, Germany; 5 Faculty of Biosciences, University Heidelberg, Heidelberg, Germany; 6 German Cancer Research Center (DKFZ), Molecular Oncology of Gastrointestinal Tumors, Im Neuenheimer Feld 280, D-69120 Heidelberg, Germany; 7 University Hospital Heidelberg, Department of Surgery, Im Neuenheimer Feld 110, D-69120 Heidelberg, Germany; 8 Present Address: Biopharmaceutical New Technologies (BioNTech) Corporation, Mainz, Germany

**Keywords:** Radiotherapy, Pancreatic cancer, Tumour immunology, Radiotherapy, Pancreatic cancer

## Abstract

Pancreatic ductal adenocarcinoma (PDA) is highlighted by resistance to radiotherapy with the possible exception of hypofractionated irradiation. As single photon doses were reported to increase immunogenicity, we investigated dose-dependent irradiation effects on clonogenic survival, expression of immunologically relevant cell surface molecules and susceptibility to cytotoxic T cell (CTL) mediated killing using a murine PDA cell line. Clonogenicity decreased in a dose-responsive manner showing enhanced radioresistance at single photon doses below 5 Gy. Cell cycle analysis revealed a predominant G2/M arrest, being most pronounced 12 h after irradiation. Polyploidy increased in a dose- and time-dependent manner reaching a maximum frequency 60 h following irradiation with 10 Gy. Irradiation increased surface expression of MHC class I molecules and of immunological checkpoint molecules PDL-1 and CD73, especially at doses ≥ 5 Gy, but not of MHC class II molecules and CXCR4 receptors. Cytotoxicity assays revealed increased CTL lysis of PDA cells at doses ≥ 5 Gy. For the PDA cell line investigated, our data show for the first time that single photon doses ≥ 5 Gy effectively inhibit colony formation and induce a G2/M cell cycle arrest. Furthermore, expression levels of immunomodulatory cell surface molecules became altered possibly enhancing the susceptibility of tumour cells to CTL lysis.

## Introduction

Despite application of continuously evolving treatment techniques and their individual escalation, prognosis for patients with PDA has remained dismal^1^. The role of radiotherapy in the multimodal treatment approach of PDA has been controversially discussed. In fact, due to its intrinsic properties this tumour entity is generally considered as highly radioresistant and non-immunogenic^2,3^. Recently, several trials have identified hypofractionated photon dose regimens to be associated with increased local control and thus potentially also with survival rates^4–7^. In conventional radiation biology, the use of increased ablative single photon doses commonly overcomes tumour-intrinsic radioresistance by inhibiting repopulation of tumour cells and repair of DNA damage that occur during normofractionated radiotherapy.

Several preclinical and clinical trials found increased single photon doses capable of eliciting immunological effects turning irradiated tumours and their stroma into pharmacologically targetable compartments^8^. Regarding PDA, only few preclinical studies have revealed immune stimulatory radiogenic effects so far; the same holds true for studies investigating the clinical benefit of radiotherapy approaches combined with immune checkpoint blockade^9–11^. Currently, there is no consensus about the appropriate photon doses that should be applied in order to induce immunomodulatory alterations in addition to merely anti-proliferative effects. While most of the clinical data available were generated using normo- and moderately hypofractionated photon doses, several authors have suggested that fractionation with “high doses” to be most effective with respect to immunomodulation in pre-clinical settings^8,9,12,13^. On the contrary, single “very high” radiosurgical doses have been repeatedly found to impair immunological responses - most likely due to their ablative cytotoxic effects^12,14^. In this context it has been suggested that single doses above 12–18 Gy (depending on cancer type) should not be exceeded as DNA exonuclease Trex1 was reported to be induced resulting in cytosolic DNA degradation thereby reducing dendritic cell activation and CD8 + T cell priming^8,14^.

Independent of the current uncertainty regarding the optimal dose application it is known that photon irradiation can exert stimulatory as well as inhibitory immunological effects, depending on the intrinsic properties of both tumour cells and stroma. These properties comprise tumour entity-associated mutational burden patterns resulting in the generation of immunogenic neo-epitopes, as well as stromal features affecting accessibility for immune cells, and further parameters^2,3,15^.

In the present study, we correlate dose-dependent radiosensitivity with radiogenic immune alterations, in a preclinical murine PDA model.

## Results

### PDA30364/OVA cells show dose-dependent sensitivity to ionizing photon radiation

**Figure 1:**
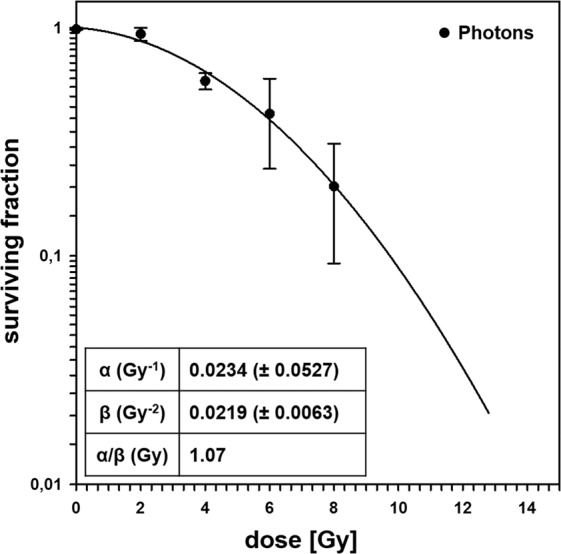
Radiation survival curve of PDA30364/OVA cells. Colony formation assay was performed applying 2, 4, 6 and 8 Gy photon irradiation. Using triplicates of each dose, experiments were performed independently on two different days. Mean values ± SD of independent experiments are shown.

In order to determine the effect of irradiation on the clonogenicity of PDA30364/OVA cells, we performed conventional clonogenic survival experiments. PDA30364/OVA cells were responsive to single doses of photon radiation in a dose-dependent manner (Fig. 1). According to the linear-quadratic model, surviving fractions for irradiation doses of 1, 3, 5, or 10 Gy were 0.956, 0.766, 0.515, and 0.089, respectively. α/β-ratio was calculated to be 1.07.

**Figure 2:**
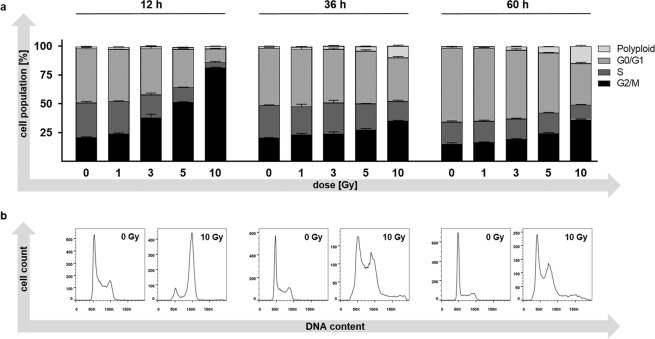
Cell cycle analysis of PDA30364/OVA cells after photon irradiation. (**a**) Quantification of cell cycle stages and of polyploidy within PDA30364/OVA cells 12, 36 and 60 h after irradiation with single doses of 1, 3, 5 or10 Gy. DNA content was determined by propidium iodide staining followed by flow cytometric analysis. Results of one out of two experiments are shown. Samples were measured in technical triplicates, presented as mean ± SD. (**b**) Exemplary histograms showing cellular DNA contents after irradiation with 10 Gy compared to control (0 Gy) 12, 36 and 60 h following irradiation.

Cell cycle analyses were performed to investigate radiation-associated alterations in the composition of the cell cycle stages among irradiated cells (Fig. 2). Determination of the DNA content by propidium iodide staining revealed the absence of a G1/S (2 N) cell cycle arrest but showed a dose-dependent accumulation of PDA30364/OVA cells within the G2/M (4 N) phase. This G2/M cell cycle arrest was most pronounced 12 h after irradiation with frequencies ranging from 21% in unirradiated cells up to 81% in cells irradiated with 10 Gy (Fig. 2a). The irradiation-induced alterations in the distribution of cell cycle stages were incompletely reversed after 36 and 60 h, with the exception of an increased fraction of polyploid (>4 N) cells, especially in the 5 and 10 Gy groups (Fig. 2a,b). Histograms of all experimental set-ups are shown in Supplementary Fig. S1.

Cell death via apoptosis/necrosis of PDA30364/OVA cells 12, 36 and 60 h following irradiation was determined by Annexin V/7-AAD staining (Supplementary Fig. S2). PDA30364/OVA cells exhibited a relative resistance to radiation-induced apoptosis/necrosis. Only irradiation with the maximal dose of 10 Gy could increase the fraction of total apoptotic/necrotic cells (Annexin V + /7-AAD- and Annexin V + /7-AAD+) compared to control (0 Gy). The effect was most prominent 60 h following irradiation, increasing the fraction of total apoptotic/necrotic cells from 1.7% in the control (0 Gy) to 28.4% in the 10 Gy group. This fraction was governed by late apoptotic/necrotic cells (Annexin V + /7-AAD+).

### Photon radiation increases surface expression of immunomodulatory molecules by PDA30364/OVA cells

**Figure 3:**
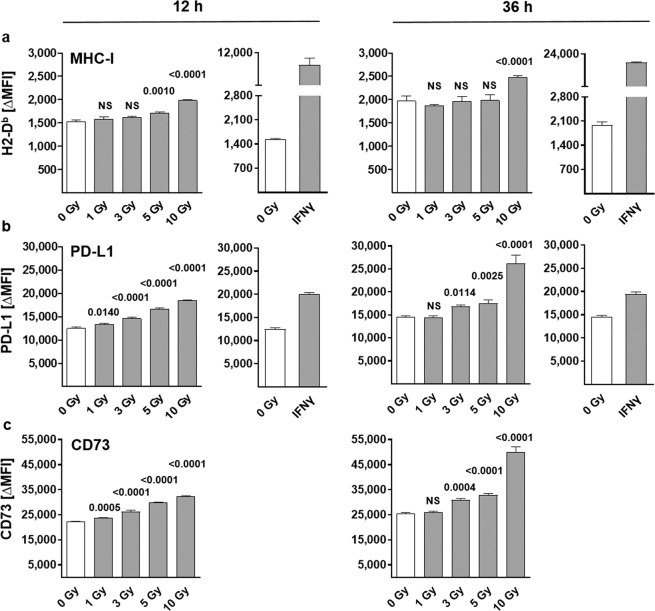
Cell surface expression of immunomodulatory molecules on irradiated PDA30364/OVA cells. Flow cytometric analysis of (**a**) MHC-I (H2-D^b^), (**b**) PD-L1 and (**c**) CD73 surface expression 12 and 36 h after irradiation with single doses of 1, 3, 5 or10 Gy. (a-c) Alteration of background-/autofluorescence was determined for each irradiation dose and time point applying combined fluorescence minus one (FMO) and isotype controls. Depicted are ΔMFI (MFI stained sample – MFI FMO/isotype control) values to compensate for dose- and time-dependent variations in background- and autofluorescence. Results of one representative experiment, performed in technical triplicates, is presented as mean ± SD and was analysed by repeated measures one-way ANOVA with correction for multiple comparison by Dunnett’s method. Each treatment was compared to control (0 Gy) and multiplicity adjusted P values are shown, α = 0.05. Inducibility of H2-D^b^ and PD-L1 expression was controlled by treatment with murine interferon-gamma (IFNγ) (20 U/ml).

Next, we performed flow cytometric analysis to investigate the effect of photon irradiation on the expression of cell surface molecules involved in immune function. Increasing irradiation doses resulted in enhanced expression of both inhibitory immune checkpoint molecules PD-L1 and CD73, as well as the T cell epitope presenting MHC class I molecule H2-D^b^ on the surface of PDA30364/OVA cells within 12 h (Fig. 3a–c, left).

Interestingly, a further increase in expression of both PD-L1 and CD73 molecules was observed 36 h after irradiation with the maximal dose of 10 Gy, while irradiation with 3 and 5 Gy increased cell surface expression of these molecules only moderately (Fig. 3b,c right). Irradiation effects on H2-D^b^ surface expression appeared generally milder, showing a slight dose-dependent increase in expression at the 12 h time point; however, only irradiation with 10 Gy was able to stably increase the expression over 36 h while lower radiation doses did not yield any significant increase anymore (Fig. 3a left and right, respectively). Notably, both PD-L1 and CD73 expression levels were already high in non-irradiated samples compared to MHC-I surface expression (see also Supplementary Fig. S3). Inducibility of PD-L1 and MHC-I surface expression on the tumour cells was controlled by IFNγ treatment (Fig. 3a,b, Supplementary Fig. S4). There was no irradiation effect observed on the surface expression of the MHC class II molecule I-A^b^. The same held true for surface expression of the CXCR4 receptor known to be involved in tumour growth and metastatic spread of various tumour entities including PDA^16,17^ (Supplementary Fig. S5). Histograms for all experimental set-ups are shown in the Supplementary Figs. S3–S5.

To investigate whether the increased cell surface expression of immunomodulatory molecules indeed resulted from *de novo* transcription, we performed quantitative PCR 12 and 36 h following irradiation (Supplementary Fig. S6). Dose-dependent changes in PD-L1 gene expression followed a similar trend as the radiogenic alteration of PD-L1 surface expression, although not statistically significant. Similar changes were observed for MHC-I (H2-D^b^) gene expression, while the expression profile of CD73, in contrast to its protein levels, showed no dose-dependency on transcriptional level.

Interestingly, the CTL line employed for functional testing of radiogenic immune sensitization of tumour cells showed surface expression of programmed death receptor protein 1 (PD-1) (Supplementary Fig. S7), thus enabling target cell interaction via the PD-1/PD-L1 axis.

### Photon irradiation enhances susceptibility of PDA30364/OVA cells to CTL lysis

**Figure 4:**
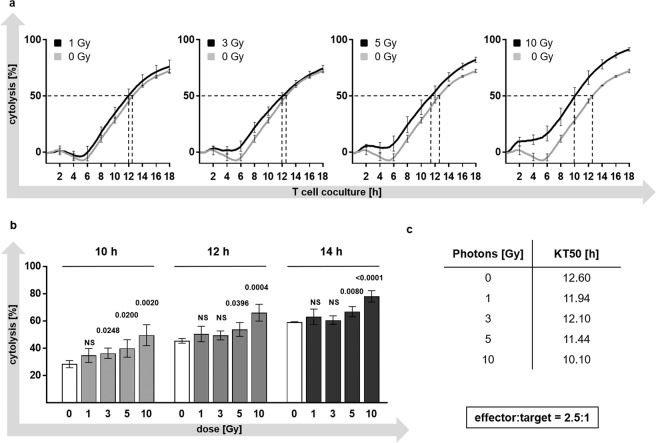
Increased susceptibility of PDA30364/OVA cells to CTL lysis following photon irradiation. (**a**) Cytolysis of PDA30364/OVA cells was monitored by measuring impedance which is proportional to the number of adherent cells. The mean decrease in impedance of wells containing PDA30364/OVA cells upon CTL co-culture relative to the mean impedance of wells containing tumour cells without CTLs was calculated and expressed as cytolysis [%] for each irradiation dose. The effector to target cell ratio was 2.5:1 and cytolysis during co-culture was monitored for at least 18 h. (**b**) Tumour cell lysis 10, 12 and 14 h after CTL co-culture for each treatment condition. (**c**) Time span required by CTLs to kill 50% of target cells was expressed as „Kill-Time-50“ (KT50) for each treatment condition. Representative results of one out of 3 experiments measured in 3–4 replicate wells are presented as mean ± SD and were analysed by two-tailed *t* test with correction for multiple comparison by Holm-Bonferroni method. Multiplicity adjusted P values are shown, α = 0.05.

In order to examine whether photon irradiation would sensitize PDA30364/OVA to CTL mediated killing we performed functional assays. Thus, PDA30364/OVA cells were irradiated with single doses of 1, 3, 5 or 10 Gy and cultured with or without ovalbumin specific CTLs 36 h later. To determine the relative extend of CTL mediated tumour cell killing for each irradiation dose the percentage cytolysis was calculated. Therefore, the decrease in cell index (representing the number of adherent cells) of irradiated cells co-cultured with CTLs was compared to the cell index of irradiated cells cultured without CTLs and was expressed as percentage cytolysis (Fig. 4a) (see material and methods for formula). Compared to the unirradiated control, single photon doses of 1, 3, 5, and 10 Gy increased the susceptibility of PDA30364/OVA to CTL lysis in a dose-dependent manner (Fig. 4a,b). Regarding irradiation with 5 and 10 Gy, enhanced susceptibility was reflected by earlier onset of cytolysis and a further constant, significant increase in cytolysis over 18 h following CTL co-culture. However, differences in cytolysis among cells treated with 1 or 3 Gy compared to untreated target cells remained insignificant over a time period of 18 h (Fig. 4a,b). To quantify the effects of irradiation-induced enhancement in CTL-susceptibility, we determined the time span required by CTLs to kill 50% of irradiated target cells expressed as “Kill-Time-50” (KT50) (Fig. 4c). KT50 reduction was most distinct after irradiation with a single dose of 10 Gy and reached 19.8% reduction in comparison to the untreated control. Specificity of the CTL line was verified by co-culture with parental PDA30364 cells devoid of OVA expression, resulting in lack of target cell recognition (Supplementary Fig. S7).

## Discussion

The presented study demonstrates dose-dependent radiation-responsiveness of a *KRAS*-driven PDA cell line in conventional radiation biology endpoints such as clonogenicity and in the current concept of irradiation-induced tumour cell immunogenicity. Notably, single photon doses between 1 and 3 Gy, as generally administered in normofractionated regimens in clinical routine, induced mostly limited anti-proliferative and immunological effects on PDA30363/OVA cells, while single doses beyond 5 Gy, as commonly used in hypofractionated regimens, considerably depressed tumour cell clonogenicity and enhanced expression of immunomodulatory cell surface molecules. Overall, in a functional setting, raising single doses above 5 Gy led to a net sensitization of PDA30364/OVA to CTL lysis.

Photon radiotherapy has been applied in many multimodal treatment regimens for decades; however, proof of clinical benefit for this approach in PDA is still missing. In fact, only few guidelines and recommendations contain radiotherapy as a standard for primary care of PDA patients^18,19^. In addition to high treatment toxicities, this reluctance is due to intrinsic and acquired radioresistance which is commonly observed both in preclinical and clinical settings^2,20^. Mechanisms associated with the radioresistant phenotype of PDA include modifications in DNA damage response, DNA repair machinery and cell cycle checkpoint controls, as well as the hypoxic environment within the tumour and the activation of stellate cells leading to fibrosis^2^.

Here we investigated the radiation-responsiveness of an OVA expressing PDA cell line carrying defined activating Kras^G12D/+^ and p53^R172H/+^ loss of function mutations while exhibiting similar histopathology to human PDA with duct-like structures and desmoplastic stroma when transplanted s.c. into immunocompetent mice^21^. In fact, *KRAS* mutation being a main driver of increased proliferation and suppression of programmed cell death has been described in over 90% of PDA^22,23^, while mutation or deletion of TP53 has been found in over 50–75% of PDA^24^. Therefore, the investigated PDA cell line is likely to be characterized by intrinsic radioresistance and highly representative for an *in vitro* analysis of clinical *in vivo* scenarios.

Opposed to commonly reported tumour cells of various entities^25^, single photon doses which are typically administered clinically (1.8–3 Gy) only mildly depressed tumour cell clonogenicity of PDA30364/OVA cells with a surviving fraction (SF) of 0.87 at 2 Gy (SF2) and an α/β-ratio of 1.07. Despite the great inter-group variation of SF2´s between cell lines of the same histological origin^25^, as well as the limitations of the α/β-ratio as a surrogate parameter for radiosensitivity^26^, the PDA cell line appeared highly radioresistant at lower dose ranges. This is in line with the finding of poor responsiveness of PDA cell lines to irradiation, as evaluated by conventional radiation biology endpoints such as clonogenicity^27–30^. *KRAS* activation in combination with loss of p53 function has been reported to account for the absence of G1/S cell cycle arrest in response to irradiation^2^. Our and published data further indicate that PDA cells might be prone to G2/M checkpoint abrogation as DNA repair is now increasingly dependent on the induction of this checkpoint. On the other hand, loss of p53 function also contributes to resistance to apoptosis^2^, which is in line with our findings that showed a moderately increased fraction of late apoptotic/necrotic PDA30364/OVA cells after 36 and 60 h following irradiation only when applying the maximal single dose of 10 Gy. Consequently, the radiation-induced distinct G2/M cell cycle arrest in PDA30364/OVA cells observed in our study might be regarded as a component of its radioresistant phenotype, providing time for DNA repair to occur to prevent mitotic catastrophe. Although not uniformly described, multiple studies have demonstrated a clear association between cellular radiosensitivity and p53 status of cell lines derived from different tissue types, thus characterizing cells expressing wild type p53 as more sensitive to ionizing irradiation^31–33^. Therefore, the results of our clonogenic survival assays, cell cycle analyses and apoptosis assays in response to single doses of photon irradiation between 1 and 10 Gy demonstrated that the suspected intrinsic radioresistance also applies to the PDA cell line investigated here. However, single doses ≥ 5 Gy, indicative of hypofractionation, still induced anti-proliferative, cytotoxic effects to a relevant degree (SF5 = 0.515).

Following the same trend, a moderate but time-persistent effect of radiation-induced immune reactivity was more prominently observed at higher radiation dose ranges greater than 5 Gy. Modestly enhanced expression levels of the immunosuppressive checkpoint molecules PD-L1 and CD73 as well as immunosensitizing MHC class I molecules, were determined with increasing radiation doses.

Notably, irrespective of the functional dichotomy of these immunomodulatory molecules we did observe a dose-dependent increase in susceptibility of PDA30364/OVA cells to CTL-mediated killing. This positive net effect of radiogenic sensitization on tumour cells resulted in a faster response to CTL recognition and a significantly increased cytolysis over a time period of 18 h. These results are in line with other reports focused on MHC class I molecule expression of a human melanoma cell line over a single fraction dose range of 1 to 25 Gy^34^. Regarding the immunosuppressive checkpoint molecules PD-L1 and CD73, their surface expression followed the same dose-dependent kinetic in PDA30364/OVA cells. Recently, Azad *et al*. showed upregulation of PD-L1 in several PDA cell lines after irradiation *in vitro* and, moreover, a strongly enhanced antitumoural immune response upon irradiation in combination with PD-L1 blockade to occur exclusively at high radiation doses (20, 12, 3 × 5 Gy) *in vivo*^9^. Similarly, Deng *et al*. found a single dose of 12 Gy to be effective not only in inducing increased PD-L1 expression on the investigated breast cancer cells itself but also on immune cells residing in the tumour microenvironment^13^.

The immunosuppressive nature of the membrane bound ectonucleotidase CD73 converting extracellular AMP to adenosine has been extensively reviewed^35,36^. Inhibition of CD73 was shown to suppress tumour growth in syngeneic mouse models^37–39^, and in fact, a first phase I/IB clinical trial investigating the preliminary activity of CD73 blocking antibody in combination with antibodies against anti PD-1 and/or adenosine A2A receptor in patients with advanced malignancies including PDA has been initiated in July 2018^40^. As a member of the damage-associated molecular patterns (DAMPs) family, ATP is increasingly released in response to cellular damage caused by ionizing irradiation^41^. Therefore, considering the dose-dependent increase of CD73 expression by the PDA cell line investigated here, we suggest CD73 as an immunological checkpoint molecule of particular significance in radio-immunotherapy approaches. However, CD73 activity may exert its full activity only in the setting of an AMP containing tumour microenvironment.

The chemokine receptor CXCR4 is expressed by a wide range of malignancies^16^ and has been found to play a pivotal role in early PDA development^42,43^. CXCR4 activation by C-X-C motif chemokine 12 (CXCL12) promotes tumour growth in para and autocrine fashion^43,44^ and fosters tumour cell migration as well as metastatic spread^17,44–46^. Since high CXCR4 expression in PDA was found to be associated with poor prognosis, CXCR4 expression has been suggested as a risk factor and prognostic marker for lymph node infiltration and distant metastasis^47^. Ionizing radiation was shown to up-regulate CXCL12/CXCR4 signalling in various tumour entities^48^. Since elevated CXCL12/CXCR4 levels in turn have been suggested to foster radioresistance^49,50^, we investigated possible enhancing effects of ionizing radiation on CXCR4 surface expression *in-vitro*, thereby evaluating the rational of combining radiotherapy with CXCR4 targeting to abrogate radio-protective functions^48^. In fact, Singh *et al*. demonstrated an improved anti-proliferative effect of gemcitabine on PDA cells *in vitro* when combined with the CXCR4 antagonist AMD3100^51^. Similarly, Feig *et al*. could restore the antitumour effects of antibody-based checkpoint inhibitors in a murine PDA model, when applied subsequently to prior administration of AMD3100^52^. In our study we could not determine any changes in the cell surface expression level of CXCR4 on *PDA30364*/OVA cells in response to irradiation with single doses from 1 to 10 Gy, which differs from results reported for mesothelioma cells^53^.

The concept of beneficial synergisms between irradiation-triggered immunomodulatory effects combined with immunotherapy has been discussed extensively during recent years^41,48,54^ and might play a pivotal role in future systemic treatment approaches.

Our findings are in line with preclinical studies showing greater immunomodulatory responses at larger doses per fraction or larger single doses, therefore favouring hypofractionation^9,13,14,34^. However, regarding the limitations of an *in vitro* setting, the optimal dose per fraction must be carefully selected as it might differently alter the tumour microenvironment in an *in vivo* setting. The significance of dose and fractionation in generating antitumour immunity was recently reviewed by Ko *et al*.^8^. Regarding the clinical application of combined radioimmunotherapy, a clinical phase II trial proposed to start in 2019 will investigate additive effects of anti-PD-L1 and anti-CD73 antibody treatment in combination with neoadjuvant chemoradiotherapy, applying stereotactic body radiation therapy with a 3 × 8 Gy fractionation schedule in luminal B breast cancer^55^. Considering PDA, the advent of stereotactic radiation techniques and the notion of their therapeutic administration in PDA patients^4–7,56^ opens promising options for the combination of such hypofractionated dose regimens with immunotherapy as documented by several ongoing clinical trials^57–60^.

Although from most of the preclinical data one could reach consensus on higher single doses or rather hypofractionation to be most effective with respect to radiation-induced immunomodulation, there still is substantial need for further systematic investigations, especially regarding tumour entities for which available data is scarce. As this is the case for PDA which is generally considered as “non-immunogenic” and mostly inert to conventional radiation treatment regimens, we investigated the potential of single photon doses ranging from 1 to 10 Gy to induce anti-proliferative as well as immunomodulatory effects on a novel PDA cell line^21^.

## Materials and Methods

### Cell lines and *in vitro* culture

PDA30364 is a pancreatic ductal adenocarcinoma cell line derived from PDA GEMM Elas-tTA/TetOCre Kras^+/G12D^ p53^+/R172H^ transgenic mice^61–63^. A PDA30364 derived stable transfectant clone expressing ovalbumin was established, designated as PDA30364/OVA throughout this paper^21^. Expression of ovalbumin was confirmed by Western blot (Supplementary Fig. S8). PDA30364 cells were cultured in DMEM medium (Thermo Fisher Scientific, Dreieich, Germany) supplemented with 10% heat-inactivated FCS, 1 mmol/L sodium pyruvate (Thermo Fisher Scientific), 100 U/ml penicillin (Thermo Fisher Scientific) and 100 μg/ml streptomycin (Thermo Fisher Scientific). PDA30364/OVA cells were cultured in the same medium, supplemented with 10 μg/ml blasticidin S HCL (Thermo Fisher Scientific).

The ovalbumin (OVA)-specific CTL line recognizing the H2-K^b^-restricted epitope OVA 257–264 (SIINFEKL)^64^ was cultured in alpha MEM (Sigma-Aldrich, St. Louis, USA) medium supplemented with 10% heat-inactivated FCS, 2.5% (v/v) supernatant of concanavalin A stimulated rat spleen cell cultures, 12.5 mmol/L methyl-α-D-mannopyranoside (Thermo Fisher Scientific), 100 U/ml penicillin (Thermo Fisher Scientific), 100 μg/ml streptomycin (Thermo Fisher Scientific), 2 mmol/L L-Glutamine (Thermo Fisher Scientific) and 0.1% 2-mercaptoethanol (Thermo Fisher Scientific). Tumour cells were grown in T75 cell culture flasks (TPP, Trasadingen, Switzerland) and passaged every 3 days. CTLs were expanded in 24-well plates (TTP) by weekly restimulation as described^64^. All cell lines were cultured at 37 °C/5% CO2.

### Photon radiotherapy

Photon irradiation was performed with a biological cabinet X-ray irradiator XRAD 320 (Precision X-ray Inc., N. Branford, USA) with a dose rate of 0.96 Gy/min for clonogenic survival assays and with a Gammacell 40 Exactor (Best Theratronics, Ottawa, Canada) with a dose rate of 0.91 Gy/min for functional analyses of radiogenic effects.

### Clonogenic survival assays

Cells (1 × 10^6^) were cultured in T75 flasks for 24 h prior to irradiation with single photon doses of 2, 4, 6 and 8 Gy. After 18 h of incubation, cells were seeded into 96-well plates adding 1 or 3 cells per well. After 14 days colonies where fixed with 70% ethanol followed by staining with 0.2% methylene blue (Merck, Darmstadt, Germany) for 10 min. Colonies were counted under the microscope applying a minimal threshold number of 50 cells for a colony to be considered surviving. In this format, the plating efficiency (PE) is defined by: $PE=\frac{1}{N}\times \,\mathrm{ln}\,\frac{96}{n-}$, where *N* gives number of cells seeded per well in a 96-well plate and *n*– represents the number of colony-negative wells per 96-well plate. Appropriate N was defined by pre-tests ranging between 1 and 3 cells. Cellular surviving fractions (SF) were calculated according to the formula: $SF=\frac{P{E}^{treatment}}{P{E}^{control}}$. Survival curves as well as α- and β-parameters were modelled according to the linear-quadratic model using Sigma Plot version 12.5 (SyStat Software, San Jose, USA).

### Flow cytometry

Immunofluorescence staining was performed using monoclonal antibodies shown in Supplementary Table S1. Cells were seeded in 6-well plates 24 h prior to treatment. As a positive control for MHC-I and PD-L1 induction, 20 U/ml murine interferon-gamma (Thermo Fisher Scientific) was administered to otherwise non-treated cell cultures 36 h prior to analysis. Twelve and 36 h after irradiation with 1, 3, 5 or 10 Gy, cells were harvested and washed with PBS (Sigma-Aldrich) followed by incubation with Zombie Violet™ Fixable Viability dye (1:1000) (Biolegend, San Diego, USA) in a total volume of 100 μl PBS at 4 °C for 20 min for live/dead cell discrimination. Subsequently, cells were incubated with fluorochrome conjugated antibodies diluted in a total volume of 100 μl PBS (2 μg/ml) containing 5 μg/ml BSA (Sigma-Aldrich) and 2 mmol/L EDTA (Sigma-Aldrich) at 4 °C for 30 min. Respective isotype matched antibodies against irrelevant epitopes as well as fluorescence minus one (FMO) controls were included for each treatment condition. Delta median fluorescence intensity (∆MFI) was calculated for each irradiation dose and time point by subtracting the MFI of combined isotype and FMO control from the MFI values of stained samples. Acquisition was performed using a FACSCanto II or LSR Fortessa (Becton Dickinson, Franklin Lakes, USA) flow cytometer run with FACS-Diva software version 6.2 (BD Bioscience). FlowJo software version 10.4.2 (Tree Star, Ashland, USA) was used to analyse at least 20,000 events per sample.

For detection of early apoptosis and late apoptosis/necrosis cells were seeded in T25 flasks 14 h prior to treatment. Twelve, 36 and 60 h following irradiation with 1, 3, 5 or10 Gy samples were prepared according to the PE Annexin V Apoptosis Detection Kit I (BD Biosciences). Acquisition was performed on LSR Fortessa (Becton Dickinson).

### Cell cycle analysis

Cells were seeded in T75 flasks 24 h prior to treatment. Twelve, 36 and 60 h after treatment with 1, 3, 5 or10 Gy irradiation, cells were harvested and washed with PBS followed by permeabilization/fixation with ice-cold 70% ethanol and incubation at 4 °C for at least 24 h. Subsequently, cells were incubated with 200 µl of a 100 µg/ml RNase (AppliChem, Darmstadt, Germany) at room temperature for 10 min followed by staining with 5 µl of a 1 mg/ml stock of propidium iodide solution (Sigma-Aldrich) at room temperature for 24 h. Acquisition was performed with a FACSCanto II cytometer using FACS-Diva software version 6.2 (BD Bioscience). Based on the DNA content, G0/G1-, S-, or G2/M cell cycle stages as well as polyploid genotypes were determined. FlowJo software version 10.4.2 (Tree Star) was used to analyse at least 10,000 events per sample.

### Real-time cytotoxicity assay

CTL mediated killing of tumour cells was assessed using the impedance based xCELLigence Real-Time Cell Analyzer System (RTCA) (ACEA Biosciences, San Diego, USA). Eighteen h after irradiation with the respective doses (1, 3, 5 or 10 Gy), PDA30364/OVA cells were seeded into E-Plate 96 (ACEA Biosciences) at a density of 7.2 × 10^3^ cells/well. After over-night culture of tumour cells, CTLs were added at an effector/tumour cell ratio of 2.5:1 and the cell index (CI), representing the relative impedance as a measure for the number of adherent cells was determined every 5 min for at least 24 h. CI values were normalized to the time point of CTL co-culture using the RTCA Software 2.0 (ACEA Biosciences). Percentage cytolysis was calculated according to the formula: $\,Cytolysis[ \% ]=[\frac{(C{I}_{wo.CTLs}-C{I}_{w.CTLs})}{C{I}_{wo.CTLs}}]\times 100$. Standard deviation (SD) of mean CI values was calculated using error propagation formulas established by the Biostatistics Department of the DKFZ. Specificity of the OVA specific CTL line was controlled using parental PDA30364 cells. “Kill-Time-50” (KT50) was defined as time span between CTL addition and eradication of 50% of PDA30364/OVA cells.

### qPCR and western blotting

RNA isolation, quantitative PCR and Western blotting are described in the Supplement.

### Statistical analysis

For qPCR assays changes of target gene expression were normalized to the housekeeping gene Rpl19, fold changes over control (0 Gy) were calculated by ∆∆Ct method and results were consecutively log-transformed. As fold changes are right skewed, normal distribution of data can be achieved by log-transformation, making statistical testing by *t* test appropriate. Therefore, the Log_2_(FC) of target gene expression for each treatment at a given time point was tested against the hypothetical value of 0 *via* GraphPad Prism software version 7.05 (GraphPad Software, San Diego, USA) using a two-tailed one-sample *t* test and correction of P values for multiple testing was done by Holm-Bonferroni method.

For flow cytometry assays repeated measurement one-way ANOVA was used to account for experiment-wise effects. Each irradiation group was compared to control (0 Gy), using Dunnett’s method to adjust for multiple testing (many-to-one comparison) *via* GraphPad Prism software version 7.05.

For cytotoxicity assays significant differences among percentage cytolysis values obtained from unirradiated cells were compared to the ones of each irradiation dose at a given time point. Significance was determined *via* RStudio software version 1.1.463 (RStudio, Boston, USA) using a two-tailed *t* test calculated with an R code created by Annette Kopp-Schneider (DKFZ Biostatistics Department). To correct for multiple comparison we applied Holm-Bonferroni method.

## Conclusions

In conclusion, we demonstrate here that pancreatic cancer, despite its radioresistance against normofractionated photon doses, may be susceptible to photon radiation when raising single doses beyond 5 Gy *in vitro*. Radiation-induced antitumoural immunity combined with targeting of immunological checkpoint inhibitors might provide novel radioimmunotherapy strategies for successful treatment of pancreatic cancer patients. However, further studies are needed to thoroughly elucidate the relationship between radiation dose and the current concept of radiation–enhanced tumour cell immunogenicity, especially with the rise of novel immunotherapeutic targets and radiation modalities such as hadron irradiation.

## Supplementary information

Supplementary information

## Data Availability

All data generated or analysed during this study are included in this article (and its Supplementary Information files).
