# Supplementary material for: Radiation-induced alterations in immunogenicity of a murine pancreatic ductal adenocarcinoma cell line
Source: Sci Rep. 2020 Jan 20;10:686. doi: 10.1038/s41598-020-57456-2 (PMC6971029; doi:10.1038/s41598-020-57456-2)
Supplement: Supplementary file 1 — Supplementary information [file 41598_2020_57456_MOESM1_ESM.pdf]

Supplementary Material to the article

Radiation-induced alterations in immunogenicity of a murine pancreatic ductal adenocarcinoma cell line

Philipp Schröter[1,2,3,4], Laura Hartmann[1,5], Wolfram Osen[1], Daniel Baumann[6,7], Rienk Offringa[6,7], David Eisel[1,5,8], Jürgen Debus[2,3,4], Stefan B. Eichmüller[1*] and Stefan Rieken[2,3,4*]

[1] German Cancer Research Center (DKFZ), Research Group GMP & T Cell Therapy, Im Neuenheimer Feld 280, D-69120 Heidelberg, Germany
[2] University Hospital Heidelberg, Department of Radiation Oncology, Im Neuenheimer Feld 400, D-69120 Heidelberg, Germany
[3] Heidelberg Institute of Radiation Oncology (HIRO), Im Neuenheimer Feld 280, D-69120 Heidelberg, Germany
[4] Heidelberg Ion-Beam Therapy Center (HIT), Im Neuenheimer Feld 450, D-69120 Heidelberg, Germany
[5] Faculty of Biosciences, University Heidelberg, Heidelberg, Germany
[6] German Cancer Research Center (DKFZ), Molecular Oncology of Gastrointestinal Tumors, Im Neuenheimer Feld 280, D-69120 Heidelberg, Germany
[7] University Hospital Heidelberg, Department of Surgery, Im Neuenheimer Feld 110, D-69120 Heidelberg, Germany
[8] present address: Biopharmaceutical New Technologies (BioNTech) Corporation, Mainz, Germany
* SBE and SR contributed equally

Correspondence and requests for material should be addressed to SBE (s.eichmueller@dkfz.de)

Supplementary Methods

RNA isolation and quantitative PCR:

Cells were seeded in T25 flasks at 14 h prior to treatment. Twelve and 36 h after treatment with 1, 3, 5 or 10 Gy, cells were harvested, pelleted and shock-frosted with liquid nitrogen. RNA was isolated using the RNeasy Mini Kit (Qiagen, Hilden, Germany) according to manufacturer´s instructions and subjected to reverse transcription using Transcriptor First Strand cDNA Synthesis Kit (Hoffmann-La Roche, Basel, Switzerland). Gene expression was quantified using quantitative PCR. Therefore, 2 X Power SYBR® Green PCR Master Mix (Thermo Fisher), 10 ng cDNA, 400 nM of each primer pair (200 nM each) and nuclease-free water were mixed to a total volume of 20 µl. The selected genes were amplified using the ABI 7300 Real-time PCR System (Applied Biosystems, Foster City, USA) and subsequently quantified by normalization to Rpl19. Primers used for quantitative PCR are depicted in Supplementary Table S2.

Western blotting:

For the preparation of whole cell protein samples, cells were harvested, counted and resuspended in an appropriate amount of Cell Lysis Buffer (2.5×10^4 cells per µl) supplemented with 1 mM Phenylmethylsulfonylfluorid (PMSF). The samples were stored on ice for 10 min and subsequently centrifuged at 4 °C and 14,000 x g for 20 min. Supernatants were transferred into fresh tubes, mixed with 5 X protein loading buffer and heat denatured at 95 °C for 10 minutes. Whole cell protein samples were separated on a 12 % polyacrylamide gel (20 min at 80 V followed by 1.5 h at 120 V) and proteins were electro-transferred onto nitrocellulose membranes using the Trans-Blot Turbo Transfer System. The membranes were blocked with 5 % of non-fat milk in Tris-buffered saline containing 0.05 % Tween (Blocking buffer) for 1 h. Subsequently, the membranes were incubated overnight at 4 °C with an OVA specific antibody diluted 1:1000 in blocking buffer. Next, membranes were incubated at RT for 1 h with a horseradish peroxidase (HRP) conjugated secondary antibody diluted 1:5000 in blocking buffer. Protein signals were detected by adding ECL Prime Western Blotting Detection Reagent directly on the membrane and exposing blots to a charge-coupled device (CCD)-camera. Afterwards the membranes were incubated at RT for 1 h with an actin specific antibody diluted 1:10000 in blocking buffer. Subsequently, the membranes were incubated a second time at RT for 1 h with a HRP conjugated antibody diluted 1:5000 in blocking solution and analysed using the ChemiDoc™ XRS+ System after adding detection reagent. Antibodies used for Western blotting are depicted in Supplementary Table S3. Data were analysed using the Image Lab Software.

Supplementary Tables

Supplementary Table S1: Monoclonal antibodies used for immunofluorescence staining and flow cytometry

Specificity	Conjugate	Cat. No.	Manufacturer	Clone
H2-D^{b}	PerCP/Cy5.5	111517	Biolegend	KH95
I-A^{b}	APC	107613	Biolegend	M5/114.15.2
CD274 / PD-L1	Brilliant Violet 785	124331	Biolegend	10F.9G2
CD73	APC/Fire 750	127221	Biolegend	TY/11.8
CD184 / CXCR4	APC	146507	Biolegend	L276F12
CD8	PE/Cy7	100721	Biolegend	53-6.7
CD279 / PD-1	APC	135209	Biolegend	29F.1A12

Isotype controls

Specificity	Conjugate	Cat. No.	Manufacturer	Clone
Isotype Ctrl.	PerCP/Cy5.5	400337	Biolegend	MPC-11
Isotype Ctrl.	APC	400611	Biolegend	RTK4530
Isotype Ctrl.	Brilliant Violet 785	400647	Biolegend	RTK4530
Isotype Ctrl.	APC/Fire 750	400455	Biolegend	RTK2071
Isotype Ctrl.	APC	400611	Biolegend	RTK4530

Supplementary Table S2: Primers used for quantitative PCR

Target	Primer	Sequence (5'-3')	Product size [bp]	Efficiency(*)	Source
Rpl19	Rpl19_fw69	TACCGGGAATCCAAGAAGATTGA	89	98.1	PrimerBank ID 226958656c3
	Rpl19_rev70	AGGATGCGCTTGTTTTTGAAC			
CD274	PD-L1_fwd	TCGCCTGCAGATAGTTCCC	133	94.6	this paper
	PD-L1_rev	TGACGTTGCTGCCATACTCC			
CD73	NT5E_fwd	GCATTCCTGAAGATGCGACC	91	100.7	this paper
	NT5E_rev	ATCGTTCTCCCGAGTTCCTG			
MHC-I (H2-D1)	H2-D1_fwd	GTGCTGCAGAGCATTACAAGG	110	91.6	this paper
	H2-D1_rev	TGCCTTTGGGGAATCTGTGC			

(*)All primers used for qPCR were tested for their amplification efficiency using a standard curve that is based on a dilution series of a cDNA sample. The efficiency was calculated using the slope of the standard curve and the following formula: Efficiency = (10(-1/slope)-1)*100

Supplementary Table S3: Antibodies used for Western blotting

Antigen	Reactivity	Coupled	Dilution	Cat. No.	Manufacturer	Clone	Isotype	Organism
Actin	mouse	-	10000	691001	MP Biomedicals	C4	IgG1	mouse
IgG	mouse	HRP	5000	sc-2005	Santa Cruz Biotechnology	polyclonal	-	goat
Ovalbumin	chicken	-	1000	sc-65984	Santa Cruz Biotechnology	3G2E1D9	IgG1	mouse

Supplementary Figures

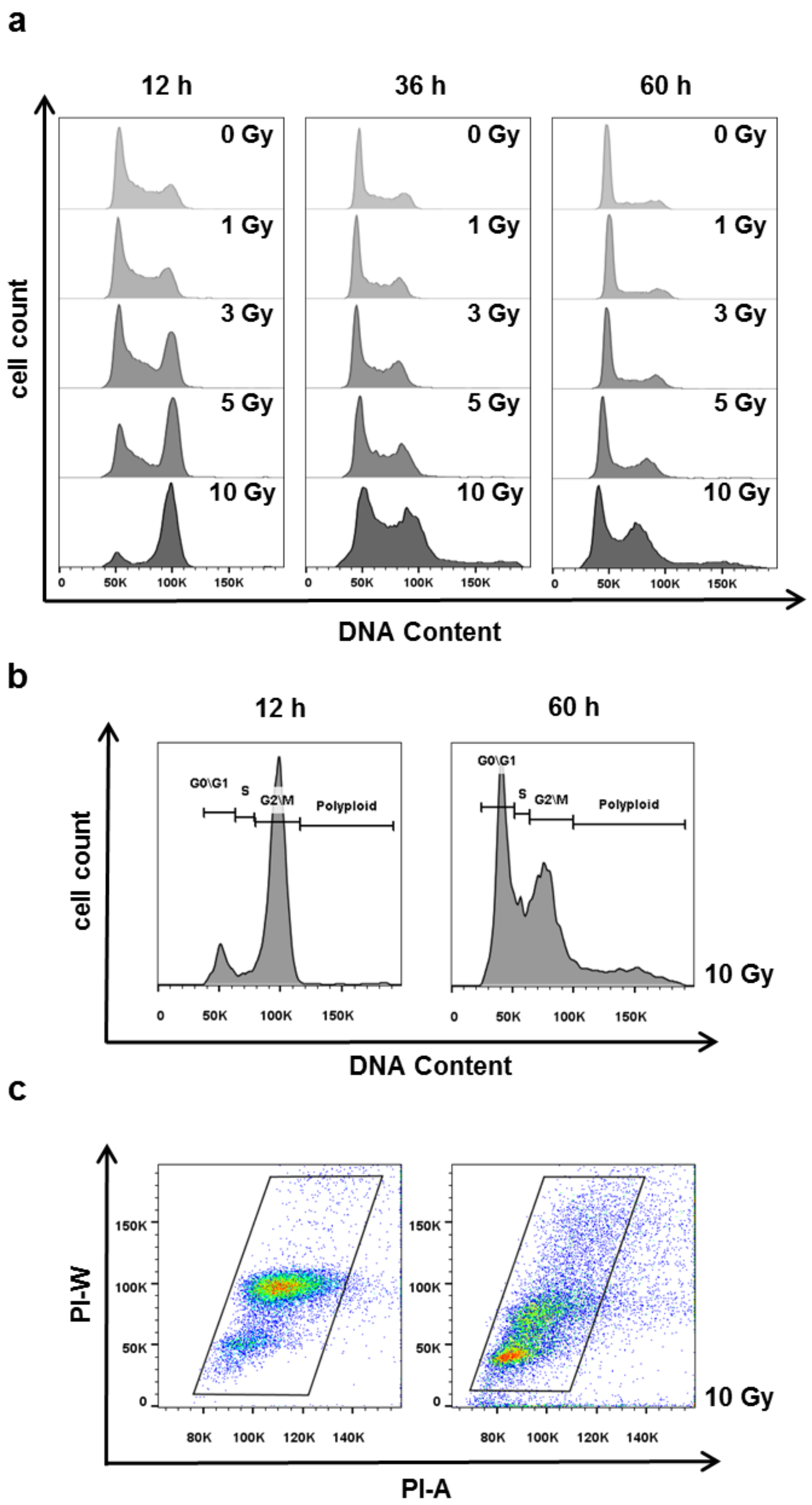

Supplementary Figure S1. Cell cycle analysis of PDA30364/OVA cells after photon irradiation. (a) DNA Quantification of PDA30364/OVA cells 12, 36 and 60 h after irradiation with single doses of 1, 3, 5 or 10 Gy by propidium iodide staining and consecutive flow cytometric analysis. **(b, c)** Analysis of irradiated PDA30364/OVA cells 12 h (left) and 60 h (right) after irradiation with 10 Gy. **(b)** Definition of cell cycle phases based on cellular DNA content. **(c)** Induction of polyploidy. Gating strategy: cells (FSC-A vs SSC-A) → single cells (PI-A vs. PI-W) → marker vs. count.

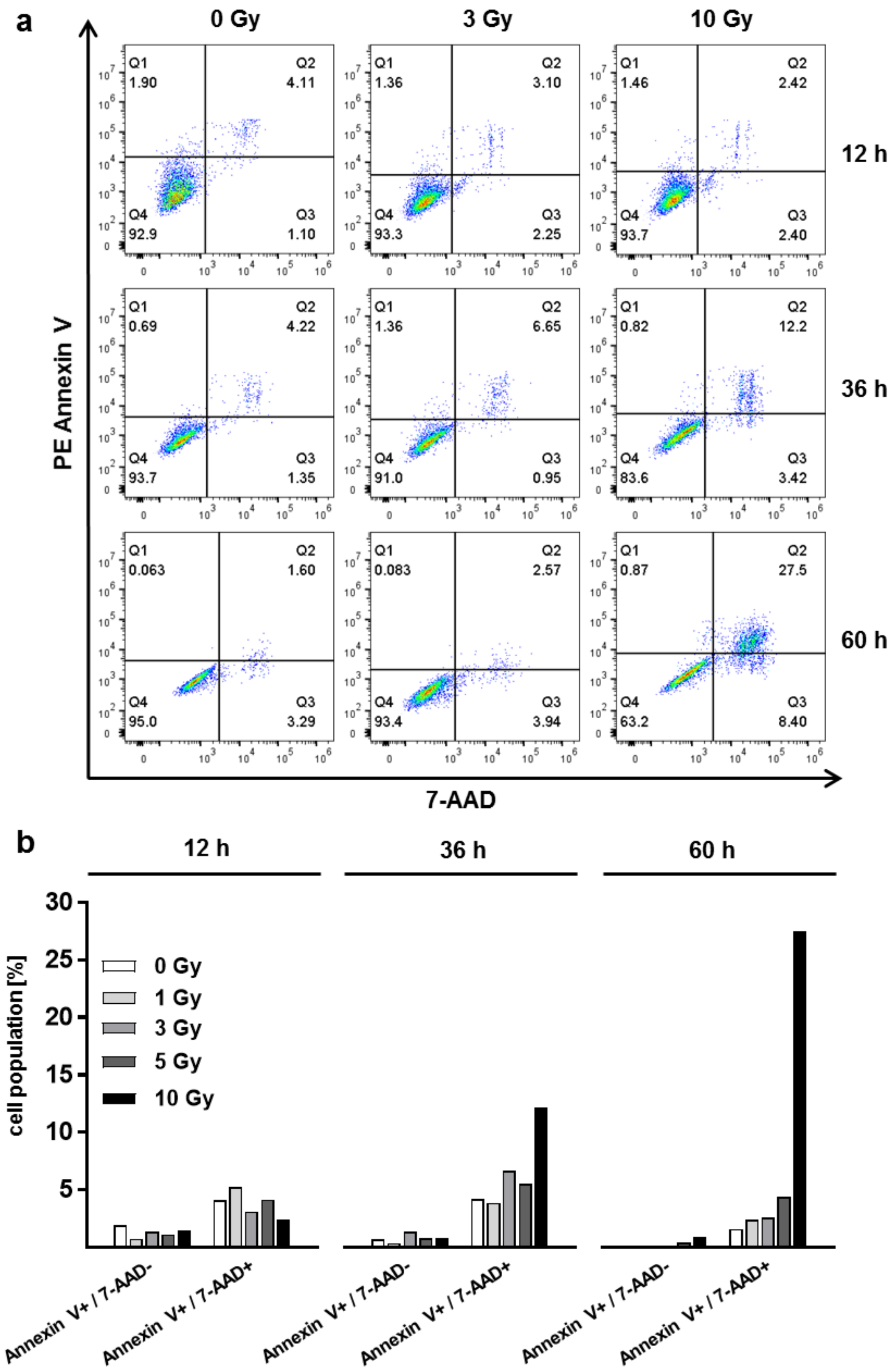

Supplementary Figure S2. Early and late apoptosis/necrosis induction in PD30364/OVA cells after photon irradiation. (a) Exemplary of PE Annexin V/7-AAD stained PDA30364/OVA cells 12, 36 and 60 h following irradiation with single doses of 1, 3 or 10 Gy. **(b)** Quantification of early apoptosis (Annexin V+ / 7-AAD-) and late apoptosis/necrosis (Annexin V+ / 7-AAD+) for all treatment conditions and time points. Gating strategy: cells (FSC-A vs SSC-A) → single cells (FSC-A vs FSC-H) → 7-AAD-A vs PE Annexin V-A.

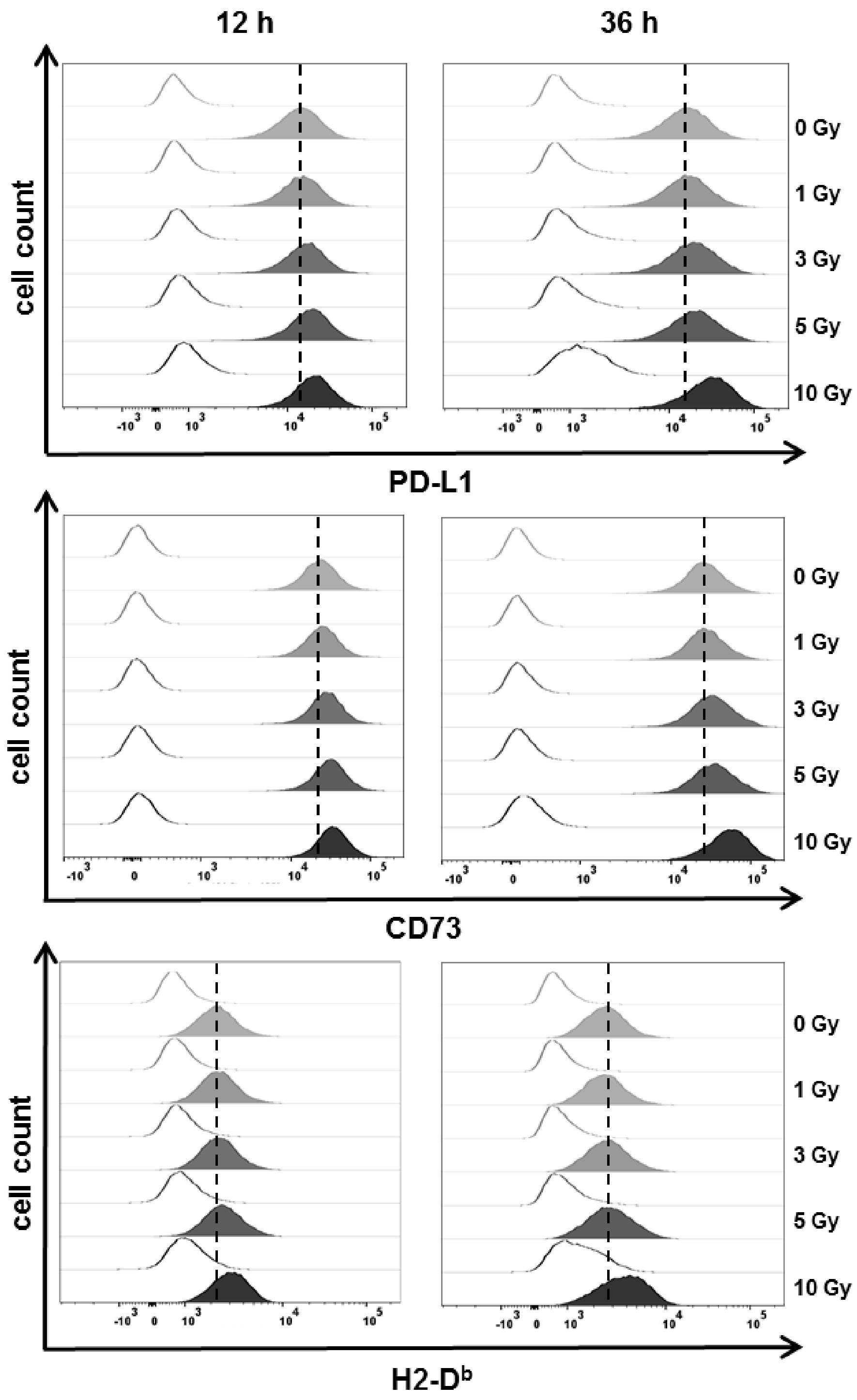

Supplementary Figure S3. Analysis of PD-L1, CD73 and MHC-I (H2-D^b) surface expression by PDA30364/OVA cells after photon irradiation. Flow cytometric analysis of PD-L1, CD73 and MHC-I (H2-D^b) surface expression 12 and 36 h after irradiation with single doses of 1, 3, 5 or 10 Gy. Gating strategy: cells (FSC-A vs SSC-A) → single cells (FSC-A vs. FSC-H) → living cells (Zombie Violet DCM vs. SSC-A) → marker vs. count. Empty histograms show combined FMO and isotype controls, filled histograms represent the stained sample.

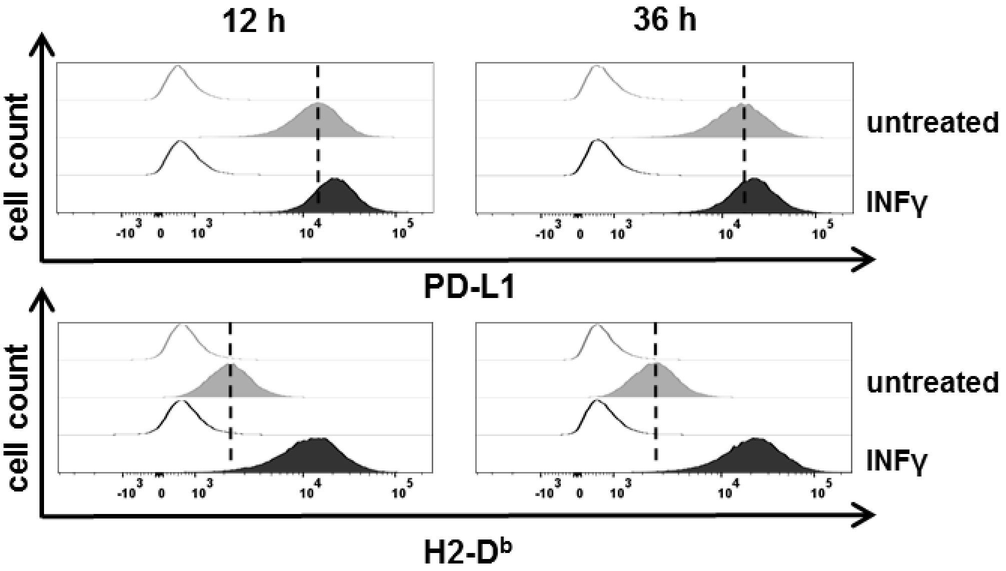

Supplementary Figure S4. Analysis of PD-L1 and MHC-I (H2-D^b) surface expression by PDA30364/OVA cells after treatment with murine IFNγ. Flow cytometric analysis of PD-L1 and MHC-I (H2-D^b) surface expression after treatment with murine IFNγ for 36 h prior to flow cytometric analysis to control for inducibility of expression. Gating strategy: cells (FSC-A vs SSC-A) → single cells (FSC-A vs. FSC-H) → living cells (Zombie Violet DCM vs. SSC-A) → marker vs. count. Empty histograms show combined FMO and isotype controls, filled histograms represent the stained sample.

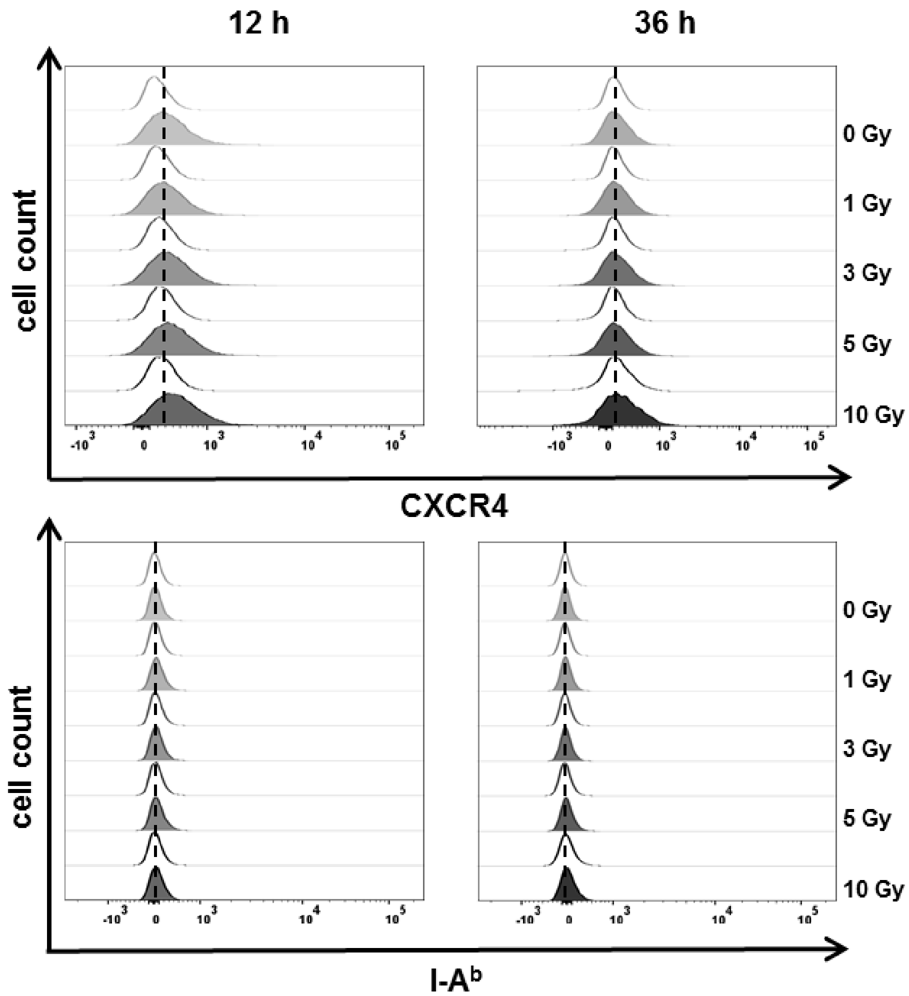

Supplementary Figure S5. Analysis of CXCR4 and MHC-II (I-A^b) surface expression by PDA30364/OVA cells after photon irradiation. Flow cytometric analysis of CXCR4 and MHC-II (I-A^b) surface expression 12 and 36 h after irradiation with single doses of 1, 3, 5 or 10 Gy. Gating strategy: cells (FSC-A vs SSC-A) → single cells (FSC-A vs. FSC-H) → living cells (Zombie Violet DCM vs. SSC-A) → marker vs. count. Empty histograms show isotype controls, filled histograms represent the stained sample.

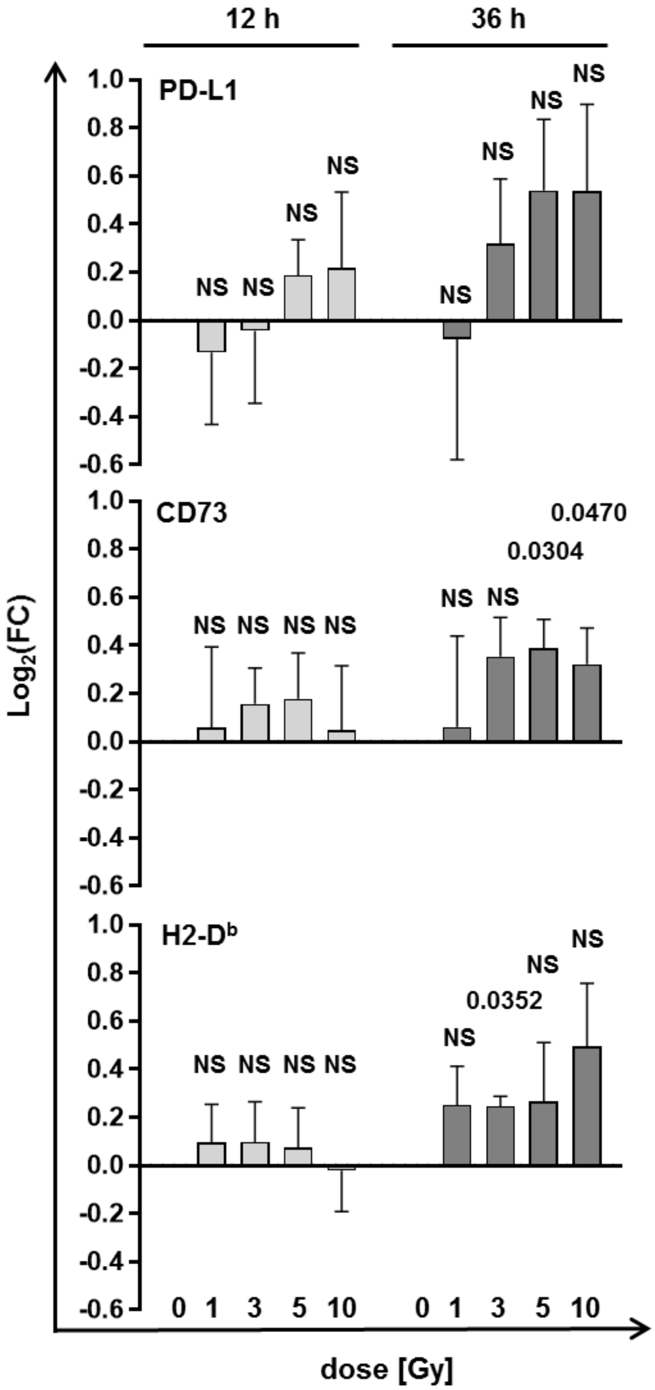

Supplementary Figure S6. Analysis of PD-L1, CD73 and MHC-I (H2-D^b) gene expression by PDA30364/OVA cells after photon irradiation. Quantitative PCR analysis of PD-L1, CD73 and MHC-I (H2-D^b) gene expression 12 and 36 h after irradiation with single doses of 1, 3, 5 or 10 Gy. Changes in target gene expression were normalized to the housekeeping gene Rpl19 and fold changes of target gene expression over control (0 Gy) were log$_2$-transformed. Mean values ± SD of 3 (H2-D^b) or 4 (PD-L1 and CD73) independent experiments each performed in technical triplicates are shown. The Log$_2$(FC) of target gene expression for each treatment and time point was tested against the hypothetical value of 0 using a two-tailed one-sample *t* test and correction of P values for multiple testing was done by Holm-Bonferroni method. Multiplicity adjusted P values are shown, α = 0.05.

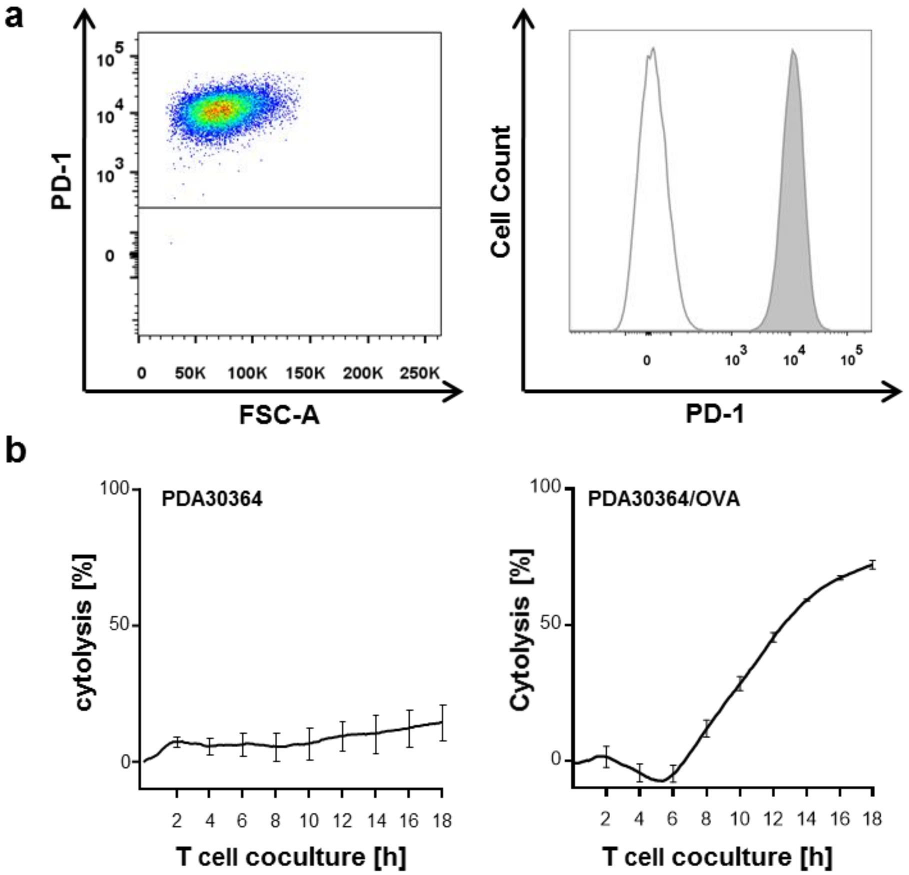

Supplementary Figure S7. Characterization of the OVA specific CTL line. (a) OVA-specific CTLs are PD-1 positive (left). Gating of positive cells was based on FMO control. Empty histogram shows FMO control, filled histogram represents the stained sample (right). Gating strategy: living cells (FSC-A vs. Zombie Violet DCM) → CD8a+ cells (FSC-A vs. CD8a) → PD-1+ cells (FSC-A vs. PD-1). **(b)** OVA-specific CTLs kill ovalbumin expressing PDA30364/OVA cells, but not parental PDA30364 cells.

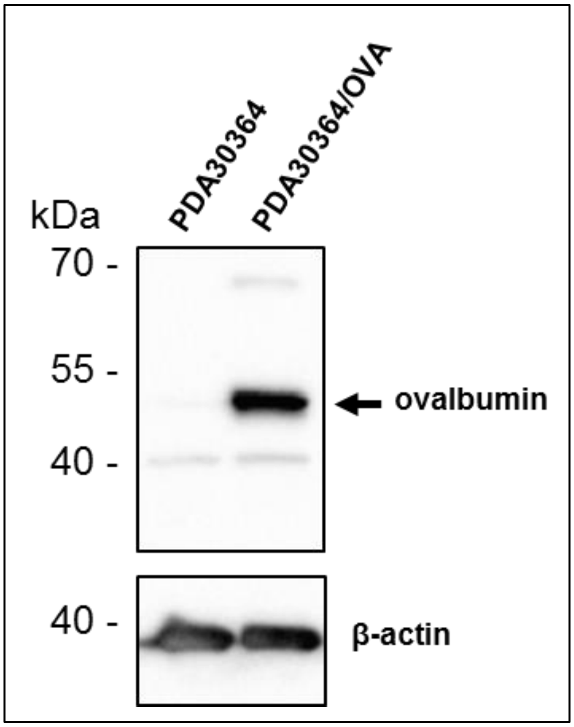

Supplementary Figure S8. Detection of ovalbumin expression in PDA30364/OVA cells. Protein extracts of the PDA30364/OVA transfectant clone and of parental PDA30364 cells were analysed by Western blotting. Molecular weight of OVA including 3X FLAG tags comprises 45.83 kDa.
